# Dasatinib in core‐binding factor acute myeloid leukemia: A promising therapeutic approach

**DOI:** 10.1002/jha2.994

**Published:** 2024-08-19

**Authors:** Shyam Srinivasan

**Affiliations:** ^1^ Department of Pediatric Oncology Tata Memorial Centre, Homi Bhabha National Institute Mumbai Maharashtra India

In the present issue of eJHaem, Rojek et al. [1] have published on the real‐world outcomes of 200 patients with core‐binding factor acute myeloid leukemia (CBF‐AML) treated between 2010 and 2023. First and foremost, I commend the authors for their work. Of several aspects discussed in this paper on real‐world outcomes of CBF‐AML, one thing stands out: the benefit of adding *KIT* inhibitors (dasatinib or midostaruin) to intensive chemotherapy in CBF‐AML. The use of tyrosine kinase inhibitors (TKI), especially dasatinib, has recently garnered interest in AML, following its remarkable success in other hematolymphoid neoplasms including CML and Ph^+^ acute lymphoblastic leukemia (ALL). Dasatinib, a multikinase inhibitor with activity on *KIT* and *SRC* activated proteins, has recently shown benefit in two other phase II studies in CBF‐AML [2, 3].

In the present study, 14 days of *KIT* inhibitors (majority receiving dasatinib) along with induction chemotherapy produced significantly superior remission rates among 21 patients with CBF‐AML compared to patients who did not receive *KIT* inhibitors during induction (95% vs. 83%, *p* = 0.01). Subsequently, patients continued to receive *KIT* inhibitors during consolidation phase, which was followed by 1‐year of maintenance therapy. The survival of these patients in the study was phenomenal, with a 3‐year event‐free survival (EFS) and overall survival (OS) of 85% and 95%, respectively. These outcomes are similar or even slightly better to the 90% remission rates and 3‐year OS of 77% reported by the CALGB group with the use of dasatinib in CBF‐AML [3]. It is noteworthy that only six of 21 patients who received a *KIT* inhibitor in the study by Rojek et al. had a *KIT* mutation. Dasatinib mainly acts by inhibiting *KIT* kinase activity, and is effective on mutations involving either the activation or the juxtamembrane loop, which are commonly seen in AML [4]. However, several studies, including the present one, show dasatinib to be effective in CBF‐AML irrespective of the *KIT* status, highlighting a mechanism of action beyond *KIT* inhibition. One reason for this could be dasatinib's ability to enhance the sensitivity of blasts to cytotoxic agents and its ability to induce differentiation of AML cells, both of which can occur irrespective of the *KIT* status [5, 6]. Additionally, regardless of mutation status, *KIT* is known to be highly expressed in CBF‐AML blasts [7].

Several aspects of the present study warrant careful consideration. First, the study included patients treated over a period of 13 years. It would be worthwhile for the authors to specify when KIT inhibitors were adopted as a treatment strategy. Additionally, an analysis should consider the prolonged timeframe, as other treatment factors may have changed considerably during this period. This would eliminate time as a source of bias in the analyses. Second, the authors mention the prognostic relevance of *KIT* was not relevant in the present study due to the fact many of the patients had *RUNX1::RUNX1T1* mutation. However, based on our experience and available literature, *KIT* can be strongly prognostic in this subgroup of CBF‐AML, especially when involving the exon 17 region [8, 9]. Therefore, this alone cannot be the reason why *KIT* was not found to be prognostic in the present study, rather the small number of patients (one‐third did not have next‐generation sequencing [NGS] done) or the addition of *KIT* inhibitors to a subset of these patients could have negated the negative prognostic impact of *KIT*. Third, the toxicity data with respect to use of dasatinib were unavailable, including information on dose modification of either *KIT* inhibitors or chemotherapy, which would be important if dasatinib were to be incorporated into routine clinical use. In this context, it is important to note that in the AMLSG trial, 33 (37%) of 89 patients discontinued dasatinib due to an adverse event [2]. Lastly, another important mutation known to co‐occur with CBF‐AML is the *FLT3* mutation. While *FLT3* mutations may themselves constitute a high‐risk group, the prognostic value of *FLT3* mutations in CBF‐AML remains to be fully elucidated. The current study did not find *FLT3* mutations to impact the outcomes; however, a meta‐analysis highlighted the heterogenous prognostic impact of *FLT3* mutations in CBF‐AML [10]. Whether TKIs such as dasatinib would impact the outcomes of these patients require larger studies.

In conclusion, dasatinib emerges as a promising agent for the treatment of CBF‐AML, demonstrating efficacy irrespective of *KIT* mutation status. The significant improvement in remission rates and OS observed in the study by Rojek et al. underscores the potential of dasatinib to enhance standard chemotherapy regimens. These findings, supported by previous phase II studies, suggest that dasatinib may play a crucial role in the therapeutic landscape of CBF‐AML. As we await the results of the ongoing phase III randomized trial (NCT02013648), it is imperative to consider dasatinib as a viable option in the treatment protocol for CBF‐AML patients, potentially setting a new standard of care and improving patient outcomes in this challenging subset of AML.

## CONFLICT OF INTEREST STATEMENT

The author declares no conflicts of interest.

## FUNDING INFORMATION

The authors received no specific funding for this work.

## ETHICS STATEMENT

The author has confirmed ethical approval statement is not needed for this submission.

## PATIENT CONSENT STATEMENT

The author has confirmed patient consent statement is not needed for this submission.

## CLINICAL TRIAL REGISTRATION

The author has confirmed clinical trial registration is not needed for this submission.

## Data Availability

Not applicable.
